# Through‐Thickness Electric Field Establishes Complex Molecular Architectures for Localized Liquid Secretion

**DOI:** 10.1002/advs.202413212

**Published:** 2025-02-06

**Authors:** Dongyu Zhang, Julia Nuijten, Jacques Peixoto, Danqing Liu

**Affiliations:** ^1^ Laboratory of Human Interactive Materials (HIM) Department of Chemical Engineering and Chemistry Eindhoven University of Technology Groene Loper 3 Eindhoven 5612 AE The Netherlands; ^2^ Institute for Complex Molecular Systems (ICMS) Eindhoven University of Technology Groene Loper 3 Eindhoven 5612 AE The Netherlands

**Keywords:** bio‐mimetics, liquid crystal networks, localized liquid secretion, responsive polymer materials

## Abstract

Localized liquid secretion, being an important process in nature such as the secretion of tears or mucus, has been an attractive point in developing biomimetic materials. However, precise localization remains challenging due to the cohesive and mobile nature of liquids. In this paper, light‐induced localized liquid secretion is demonstrated on the scale of tens of micrometers by a liquid crystal polymer coating with an alternating homeotropic‐planar alignment. The light responsiveness is achieved by the incorporation of azobenzene derivative. The localization is achieved by applying regional through‐thickness electric fields to the monomeric liquid crystals before polymerization. The polymerized coating preserves both homeotropic and planar alignment. Upon actuation, the liquid can be locally secreted from the homeotropic region while suppressed in the planar area. This method allows precise control over various secretion patterns based on different pre‐designed electrodes, which paves the way for the development of responsive devices in a multitude of fields, such as targeted drug delivery, tissue engineering, and microfluidic devices.

## Graphical abstract

A novel strategy is employed to achieve precise, localized liquid secretion from a liquid crystal polymer coating with homeotropic and planar alternating alignment. By applying a through‐thickness electric field to liquid crystal monomers, distinct regions of different alignments are formed. The coating after polymerization can reversibly secrete liquid at specific sites on demand. Moreover, complex patterns of localization can be achieved by designing the distribution of the electric field.

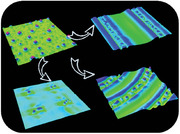

## 1 Introduction

In biological organisms, localized liquid secretion serves vital functions across a broad spectrum of processes, such as the secretion of resin by pine trees to seal wounds and protect against pathogens, or the release of enzymes in the human stomach, which occurs precisely at the site of food breakdown to facilitate digestion. Enlightened by these intriguing secreting phenomena, researchers are dedicated to boosting the development of artificial systems. Numerous rational designs aiming at the dynamic surface with fluid by tailoring the chemical composition, structural features, and material interaction have been explored.^[^ ^1^, ^2^, ^3^, ^4^, ^5^, ^6^, ^7^, ^8^, ^9^, ^10^ ^]^ However, due to the cohesive and mobile nature of liquids, mimicking locally secreting in a spatially controlled manner remains challenging. To address this issue, one promising approach is to trigger specific zones of the matrix that are filled with liquid inclusions, which typically requires the use of masks for localized activation.^[^ ^11^, ^12^ ^]^ Nevertheless, the mask‐based trigger can be complicated in practical operation, and may not always be effective. Given this, a more feasible strategy is to program the molecular architecture of the material to achieve localized liquid secretion through a uniform triggering mechanism.

Recently, our group reported a novel approach for localized liquid secretion by utilizing the anisotropic liquid crystal networks (LCNs).^[^ ^13^ ^]^ This coating self‐contains liquid and is configured to have distinct zones of smectic and isotropic phases through a two‐step polymerization process. When exposed to UV light, only the smectic phase zones will deform to locally secrete droplets. However, this method introduces some drawbacks such as the diffusion of materials in the first step of polymerization and the thermal phase separation in the second step of polymerization.^[^ ^14^ ^]^ Here in this work, we develop a new method by introducing complex molecular alignment via a patterned electric field. This method only requires one‐step polymerization, which restrains the molecular diffusion, thus enabling precise localization. The liquid crystals (LCs) are alternately aligned in homeotropic and planar configurations by regionally applying a through‐thickness electric field before polymerization. Through photopolymerization, the alternating alignment is fixed in the network. In the meantime, the liquid is included in the network via phase separation. Besides, a light‐responsive azobenzene derivative is co‐polymerized in the network to enable photo‐responsiveness. Upon exposure to UV light, the fluid is locally secreted from homeotropically aligned regions due to contraction along the thickness, which generates mechanical force to eject liquid. In contrast, secretion from the planar region is restricted. This method achieves the localization of liquid secretion as fine as 10 µm. Moreover, complex patterns of localization, including straight or circular stripes or squares are generated, which opens up their potential for precise control of smart responsive materials and devices.^[^ ^15^, ^16^ ^]^

## 2 Results and Discussion

**Figure 1:**
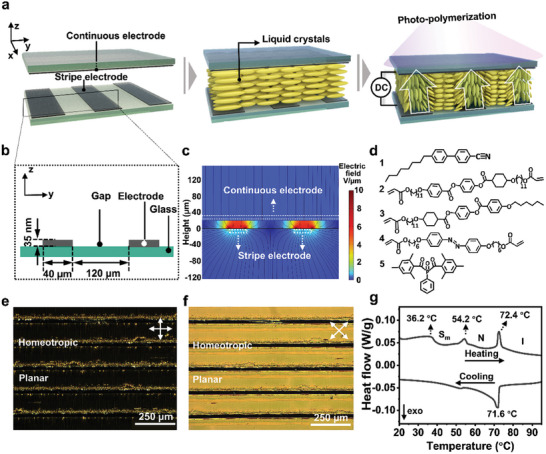
Design of the LCN polymer coating with alternating alignment. a) Scheme illustrating the fabrication of the LCN coating by aligning LCs via a patterned electric field under direct current. b) A cross‐sectional perspective of the stripe electrodes reveals their structure, with each electrode measuring 40 µm in width and separated by a 120 µm gap. c) Simulated electric field distribution from a cross‐section perspective. d) Material composition of the LCN polymer coating. Polarized optical microscopy (POM) images showing the birefringence of the LCN coating when the cross polarizer is e) 0 ° and f) 45 ° to the planer alignment direction. g) Differential scanning calorimetry (DSC) characterization for the monomer liquid crystal mixtures.

The material design is shown in **Figure** **1**a, liquid crystal monomers with desired alternating homeotropic and planar alignment are captured in LCN through photopolymerization.^[^ ^17^, ^18^, ^19^ ^]^ This hybrid alignment is achieved by combining surface anchoring alignment and electric field‐induced alignment techniques. To allow the LCs to follow the electric field, we chose molecule **1** (4′‐Octyl‐4‐biphenylcarbonitrile, 8CB) due to its positive dielectric property.^[^ ^20^, ^21^, ^22^ ^]^ It was mixed with reactive mesogens (molecules **2** and **3**) to achieve the desired smectic phase, as this phase ensures highly ordered LCs that support optimal secretion properties.^[^ ^23^, ^24^ ^]^ To endow the coating with photo‐responsiveness, we also incorporated the azobenzene derivative **4**.^[^ ^25^, ^26^ ^]^ The given mixture is initially planarly aligned via surface anchoring force. By applying a regional electric field throughout the thickness via a patterned electric field, the LCs experiencing the electric field are rotated 90° (Figure 1a,b). This patterned electric field is generated by using striped indium tin oxide (ITO) electrodes on one side and a continuous ITO layer on the other side (Figure 1c). As a result, the homeotropic and planar alternating alignment is achieved. This alignment is checked by polarized optical microscopy (POM). As shown in Figure 1e, the mesogen located between the electrodes remains black upon rotating the cross‐polarizer, while the rest of the area switches from black to yellow when the cross‐polarizer is rotated 45°(Figure 1f). It confirms the homeotropic and planar alignment at the electric field and non‐electric field zone, respectively. Subsequently, this alternating alignment is preserved by polymerizing the monomer into a network (Figure 1g; Figures S1 and S2, Supporting Information). After photopolymerization, the 8CB phase separates from the network and stays as liquid inclusions in the network.^[^ ^27^ ^]^

**Figure 2:**
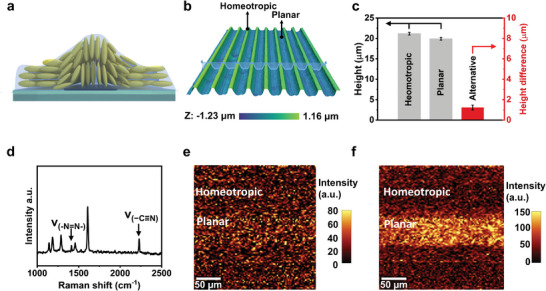
Formation of surface topography on the homeotropic‐planar alternating LCN coating. a) Schematic illustration and b) measured 3D profile image of the LCN polymer coating. c) The height of uniaxial homeotropic and uniaxial planar coatings after polymerization, as well as the height difference of the homeotropic and planar regions of alternating LCN coating. Confocal Raman Microscopy shows d) the Raman spectra and the distribution of e) azobenzene and f) 8CB in the LCN coating.

We investigated the topography of our LCN coating after polymerization by surface characterization via interferometer. The 3D surface profile image reveals an indentation in the planarly aligned region, exhibiting a distinct stripe‐shaped pattern across the surface (**Figure** **2**a,b; Figure S3, Supporting Information). We postulate this topography is formed by polymerization shrinkage or material diffusion.^[^ ^28^, ^29^, ^30^ ^]^ To analyze this, we prepared uniaxial planar and homeotropic liquid crystal polymer coatings respectively using the same liquid crystal mixture, thereby eliminating the potential influence of material diffusion and focusing exclusively on the effect of shrinkage. Initially, the coating thickness before polymerization is fixed to 20 µm. After polymerization, the uniaxial planar‐aligned liquid crystal coating exhibits a greater shrinkage of 1.27 µm in comparison to the uniaxial homeotropic‐aligned liquid crystal coating (Figure 2c). This value is comparable to the height difference (1.23 µm) between the homeotropic and planar regions measured on our alternately aligned liquid crystal coating. Furthermore, we ascertain if the material diffusion accounts for the topography by mapping the chemical distribution in the polymerized coating. As shown in Figure 2d–f, the azobenzene moieties remain relatively homogeneous after polymerization, while the 8CB diffuses to the planar region. The 8CB lateral diffusion could be driven by the faster polymerization in the homeotropic region as the dichroic azobenzene absorbs less UV light in the homeotropic region than in the planar region during photopolymerization.^[^ ^28^, ^31^, ^32^, ^33^, ^34^ ^]^ These findings confirm that the indentation in the planar region is not primarily caused by material diffusion.

**Figure 3:**
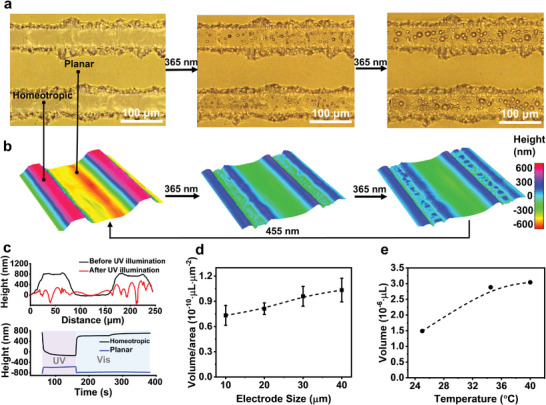
Liquid Secretion from the homeotropic‐planar alternating LCN polymer coating. a) Microscope image and b) corresponding 3D surface profile extracted via Digital Holographic Microscopy (DHM) characterization showing the localized liquid secretion before, during, and after UV illumination. c) 2D surface profile (top) and surface height change (bottom) of the LCN coating subjected to UV and blue light illumination. The volume of liquid secretion at the homeotropic area upon UV exposure changes as a function of d) the electrode size and e) the temperature of the coating substrate.

Next, we explored the liquid secretion of our LCN coating by optical microscopy and digital holographic microscopy (DHM). Upon UV illumination, azobenzene moieties isomerize from *trans* to *cis* state, resulting in the decrease of the order parameter in the network.^[^ ^35^ ^]^ This leads to anisotropic deformations with contraction along the molecular alignment direction and expansion perpendicular to the alignment direction.^[^ ^36^ ^]^ Given the geometric restriction of the substrate, the deformation is confined to the thickness direction, which eventually results in an 800 nm contraction at the homeotropic region, corresponding to 4% of the coating thickness (**Figure** **3**a–c; Videos S1 and S2, Supporting Information). Meanwhile, the adjacent planar regions expand by 200 nm in the thickness direction, driven by the x‐y in‐plane expansion from the homeotropic regions. The given deformations induce the secretion of 2.04·10^−6^ µL liquid from the homeotropic region, equivalent to ≈1% of the total liquid in the local area (Table S1, Supporting Information). The secretion is primarily attributed to the volume shrinkage occurring in the homeotropic region (Figure S4, Supporting Information). Consequently, no secretion is observed in the planar region. Upon blue light irradiation, the *cis*‐to‐*trans* back isomerization of azobenzene makes the network recover to the initial state, generating capillary suction force to reabsorb the liquid into the network (Figure 3c; Videos S1 and S2, Supporting Information). The reversible liquid secretion can be repeated for multiple cycles, indicating good stability of our coating (Figure S5, Supporting Information).

We further investigated the impact of electrode size on the actuation and secretion performance. As displayed in Figure 3d, reducing the electrode width from 40 to 10 µm, while maintaining the gap‐to‐electrode ratio, results in a corresponding decrease in secretion volume (Figure S6 and Video S3, Supporting Information). The secretion per unit area gradually decreases from 1.0·10^−10^ to 0.7·10^−10^ µL·µm^−2^ as the electrode is reduced to 10 µm. This is because the transition from homeotropic to planar region becomes more dominant, and thus the relative homeotropic region decreases (Figure S7, Supporting Information). Moreover, we analyzed the secretion performance at different temperatures. When the temperature is increased from room temperature to 35 and 41 °C, the 8CB is changed from the smectic phase to the nematic and isotropic phase, respectively, while the LCN stays in the smectic phase. This temperature‐induced phase separation between 8CB and LCN results in an increase in liquid secretion, driven by the mismatch in molecular disorder and volume expansion between 8CB and the network (Figure 3e).^[^ ^23^, ^37^, ^38^ ^]^ However, since the phase separation occurs globally, it also triggers secretion in the planar regions, causing the liquid release to lose its localized nature (Figures S8–S10, Supporting Information).

**Figure 4:**
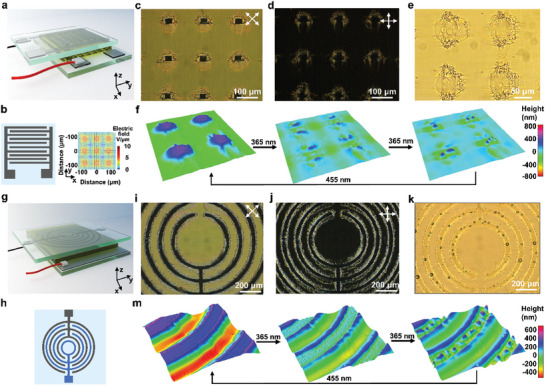
Localized liquid secretion from various patterned electric fields. Liquid secretion from square‐shaped patterns. a) The schematic diagram shows the cell configuration to obtain the square‐shaped electric field using the b) stripe‐patterned electrodes on the cover glass and bottom glass. The insert is the simulated 3D electric field distribution from the top view. POM images show the alignment of the coating with square‐shaped patterns when the cross‐polarizer is c) 45° and d) 90° to the planar alignment direction. e) Optical microscope image shows the liquid secretion from the square‐shaped patterns. f) DHM shows the 3D surface profile of the coating before, during, and after UV illumination. g) The schematic diagram shows the cell configuration to obtain the circular‐shaped electric field with the h) circular‐patterned electrode used for cover glass and a continuous electrode used for bottom glass. POM images show the alignment of the coating with circular‐shaped patterns when the cross‐polarizer is i) 45° and j) 90° to the planar alignment direction. k) Optical microscope image shows the liquid secretion from the circular‐shaped patterns. m) DHM shows the 3D surface profile of the coating, before, during, and, after UV illumination.

Beyond locally secreting from stripe‐shaped patterns, we can confine the secretion to more complicated patterns by applying different pre‐designed electric fields. As shown in **Figure** **4**a,b, to upgrade the stripe‐shaped patterns into square‐shaped patterns, we used two glass substrates with the same stripe‐shaped electrodes for the upper and bottom substrates respectively. The electrodes on the upper glass and the bottom glass were placed perpendicular to each other. When applying a direct current electric field from the bottom electrode to the top electrode, the overlapped area experiences the strongest electric field (Figure 4b). Therefore, the mesogens located at the corresponding area are rotated toward homeotropic alignment (Figure 4c,d; Figure S11, Supporting Information). After curing the coating by polymerization, the homeotropic area displays a square‐shaped protrusion on the surface. Upon illuminating the polymerized coating, the protrusions with homeotropic alignment contract into the surface and repel liquid droplets from local areas (Figure 4e,f; Video S4, Supporting Information). We further introduced curvatures to the patterns by using a circular‐shaped electrode and a continuous conductive electrode as the upper and bottom electrodes, respectively (Figure 4g,h). Upon applying an electric field, even though the mesogens between electrodes are experiencing various angles to the pattern direction, all of them can be rotated 90° as normal (Figure 4i,j). After polymerization, we observed similar contraction behavior at homeotropic areas under UV exposure, which causes droplets to be released out along the homeotropic circular patterns (Figure 4k,m; Video S5, Supporting Information).

## 3 Conclusion

In conclusion, our liquid crystal polymer coating with homeotropic‐planar alternating alignment breaks the limitation of liquid‐secreting materials, enabling localized secretion within the area of tens of micrometers. This potentially allows precise control of the fluidic environment in systems. Furthermore, various liquid crystal molecular architectures for localization are achieved, which facilitates our material's adaptation to a variety of complex application scenarios. Future efforts may focus on the localized secretion of various liquids, upon different stimuli, or even under their synergistic trigger. We anticipate that our photo‐responsive localized liquid‐secreting coating can be used for a range of abroad applications, such as microfluidic systems, microbial environmental regulation, or microscale drug delivery systems.

## 4 Experimental Section

### Materials

Low‐molecular‐mass liquid crystal **1** (4′‐Octyl‐4‐biphenylcarbonitrile, 8CB), monoacrylate molecule **3,** and azobenzene derivative **4** were purchased from Synthon Chemicals. Diacrylate molecule **2** was purchased from Syncom B.V. Photo initiator **5** (Irgacure 819) was purchased from Ciba. A typical recipe that was used in this work was a mixture that contained 54% nonreactive LC molecule **1**, 10 wt.% diacrylate **2**, 29 wt.% monoacrylate **3**, 6 wt.% azobenzene diacrylate **4**, and 1.0 wt.% photoinitiator **5**. All the liquid crystal monomer mixtures were made by dissolving them in tetrahydrofuran (purchased from Sigma–Aldrich) and then evaporating the solvent at 80 °C. Glass substrates with interdigitated indium tin oxide electrodes (IDE, active area: 1.4 × 1.0 cm) were purchased from Walthy Precision Co., Ltd.

### Sample Preparation

Coatings with smectic alignment were typically prepared by using the cell construction where one of the glass substrates has IDE and the other glass substrate has a continuous indium tin oxide conductive layer (or IDE layer). The glass substrates were cleaned by sonicating in an acetone and isopropanol bath for 20 min, respectively, and followed by UV‐Ozone treatment for 20 min. Thereafter, to offer the planar alignment to the liquid crystal mixture, a layer of polyimide (Optimer AL11254, JSR) was spin‐coated on both glass substrates. The substrates were heated on a hot plate at 90 °C for 10 min to evaporate the solvent and then cured at 180 °C for 90 min. Before using, they were gently rubbed on polyester fabric with the desired alignment direction. Subsequently, the cell was formed by gluing the treated glass substrates with glue containing spacers of 20 µm to have a controlled gap. The liquid crystal monomer mixture was filled in the cell by capillary force in its isotropic phase and then cooled down to room temperature for the planar alignment in the smectic phase. To obtain the alternating alignment, 110 volts was applied throughout the thickness by connecting one of the electrodes of the IDE and the continuous indium tin oxide layer to the cathode and anode of the direct current power supplier (SourceMeter, Keithley 2400), respectively. Next, the mixture was polymerized by a UV lamp (Omnicure EXFO S2000) at 30 °C. A 400 nm cut‐off filter (Newport FSQ‐GG400 filter) was placed between the sample and the light source to prevent premature isomerization of the azobenzene. After photopolymerization, the glass substrate with IDE layer was removed.

### Finite Element Modeling

2D and 3D multi‐physics models COMSOL Multiphysics were developed based on the electrode geometry. The conservation of currents was used to calculate the electric field distribution (E) between the designed electrodes under applied voltage difference at the boundaries:

(1) E=−∇V

(2) $$\nabla \;\left(\varepsilon_{0}\varepsilon_{r}E\right)=\;\rho_{v}$$

where V is the electric potential.ρ_*v*_ is electric volume charge density, ε_0_ is the permittivity of free space, ε_*r*_ is the relative permittivity of the material.

### Characterization

The alignment and optical properties of LCN coating were observed by a cross‐polarized optical microscope (Leica DM2700). Phase‐transition temperatures of liquid crystal monomer mixtures were measured by Differential scanning calorimetry (Q2000, TA Instruments) at a rate of 5 °C min^−1^. The thickness and surface profile of the coating was measured by interferometer (Sensofar S neox). The liquid secreted on the surface of the LCN coating was quantified by a DHM (DHM‐R, Lyncée Tec.) combined with image analysis. The Secretion volume was calculated by a custom MATLAB script based on the 3D profile of the coating measured via the DHM. The microstructure of the LCN coating was observed with a scanning electron microscope (FEI SEM Quanta 3D FEG) in secondary electron mode. Before SEM characterization, 8CB was removed from LCNs by cyclohexene for 8 h. Material distribution of the LCN coating was characterized by Confocal Raman microscope (WITec WMT 50) at room temperature using a WITec α‐300 R µ‐Raman system. Grazing‐incidence wide‐angle X‐ray scattering (GIWAXS) was performed on an instrument from Ganesha Lab. The flight tube and sample holder are all under vacuum in a single housing, with a GeniX‐Cu ultralow divergence X‐ray generator. The source produces X‐rays with a wavelength (λ) of 0.154 nm and flux of 1 × 108 p s^−1^. Scattered X‐rays were captured on a 2D Pilatus 300K detector with 487 × 619 of 172 × 172 µm^2^ pixel resolution. The sample was placed at an angle (θ) of 0.18°. The instrument was calibrated with diffraction patterns from silver behenate.

## Conflict of Interest

The authors declare no conflict of interest

## Supporting information



Supporting Information

Supplemental Video 1

Supplemental Video 2

Supplemental Video 3

Supplemental Video 4

Supplemental Video 5

## Data Availability

The data that support the findings of this study are available from the corresponding author upon reasonable request.
