# Supplementary material for: Through‐Thickness Electric Field Establishes Complex Molecular Architectures for Localized Liquid Secretion
Source: Adv Sci (Weinh). 2025 Feb 6;12(12):2413212. doi: 10.1002/advs.202413212 (PMC11948005; doi:10.1002/advs.202413212)
Supplement: Supplementary file 1 — Supporting Information [file ADVS-12-2413212-s001.docx]

Supporting information for

**Through-thickness Electric Field Establishes Complex Molecular Architectures for Localized Liquid Secretion**

Dongyu Zhang^1, 2^, Julia Nuijten^1^, Jacques Peixoto^1, 2^, Danqing Liu^1, 2*^

D. Zhang, J. Nuijten, J. Peixoto, Prof. D. Liu

Laboratory of Human Interactive Materials (HIM), Department of Chemical Engineering and Chemistry, Eindhoven University of Technology, Groene Loper 3, 5612 AE Eindhoven, the Netherlands

D. Zhang, J. Peixoto, Prof. D. Liu

Institute for Complex Molecular Systems (ICMS), Eindhoven University of Technology, Groene Loper 3, 5612 AE Eindhoven, the Netherlands

*Corresponding to: danqing.liu@tue.nl

CONTENTS:

1. SEM images

2. GIWAXS measurement of the LCN coating

3. 2D surface profile of the LCN coating

4. Comparision of the secretion based on different uniform alignment

5. Stability of the LCN coating

6. Influence of electrode size on liquid secretion

7. Influence of electrode size on transition area

8. Influence of temperature on liquid secretion

9. Optical microscope image of liquid secretion at 41°C

10. Evaluation of the thermal effect of UV illumination

11. Simulated square-shaped electric field distribution


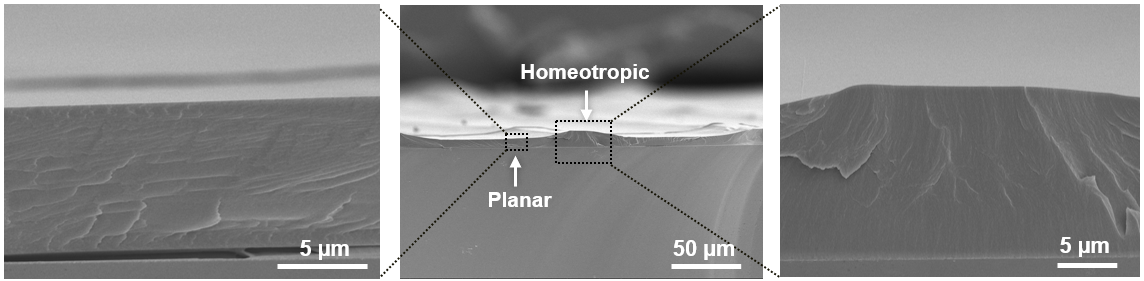


**Figure S1. SEM images of our LCN coating from a cross-sectional perspective after removing 8CB.** Middle: an overview of the coating with homeotropic-planar alternating alignment. Left: zoomed-in of the planarly aligned region. Right: zoomed-in of the homeotropic region.


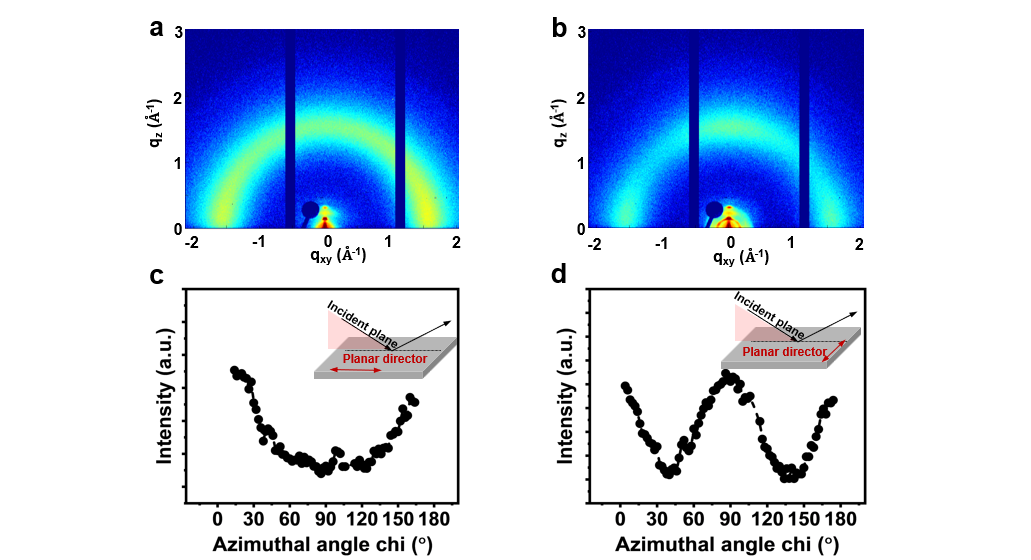


**Figure S2. Grazing-incident wide-angle X-ray scattering (GIWAXS) characterization of the homeotropic-planar alternating LCN coating.** 2D GIWAXS patterns of the coating when the plane of incidence is a) parallel to and b) perpendicular to the coating’s planar alignment director, respectively. The c) and d) are azimuthal profiles of a) and b), respectively (q=1.2-1.8 Å^-1^).


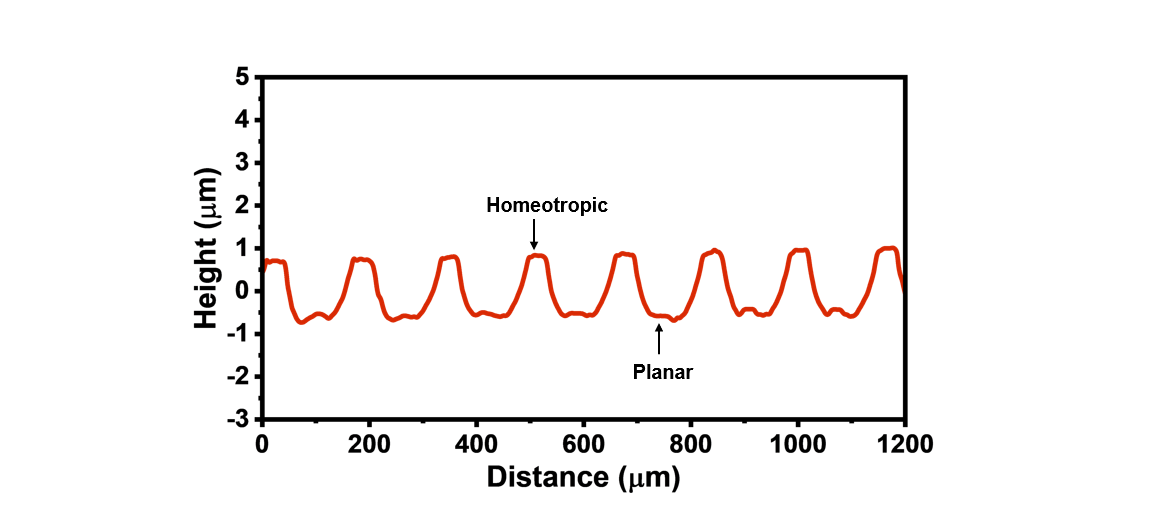


**Figure S3. The 2D surface profile of the alternately aligned coating after photopolymerization.** The average height difference between the homeotropic and planar regions is 1.23±0.23 µm.


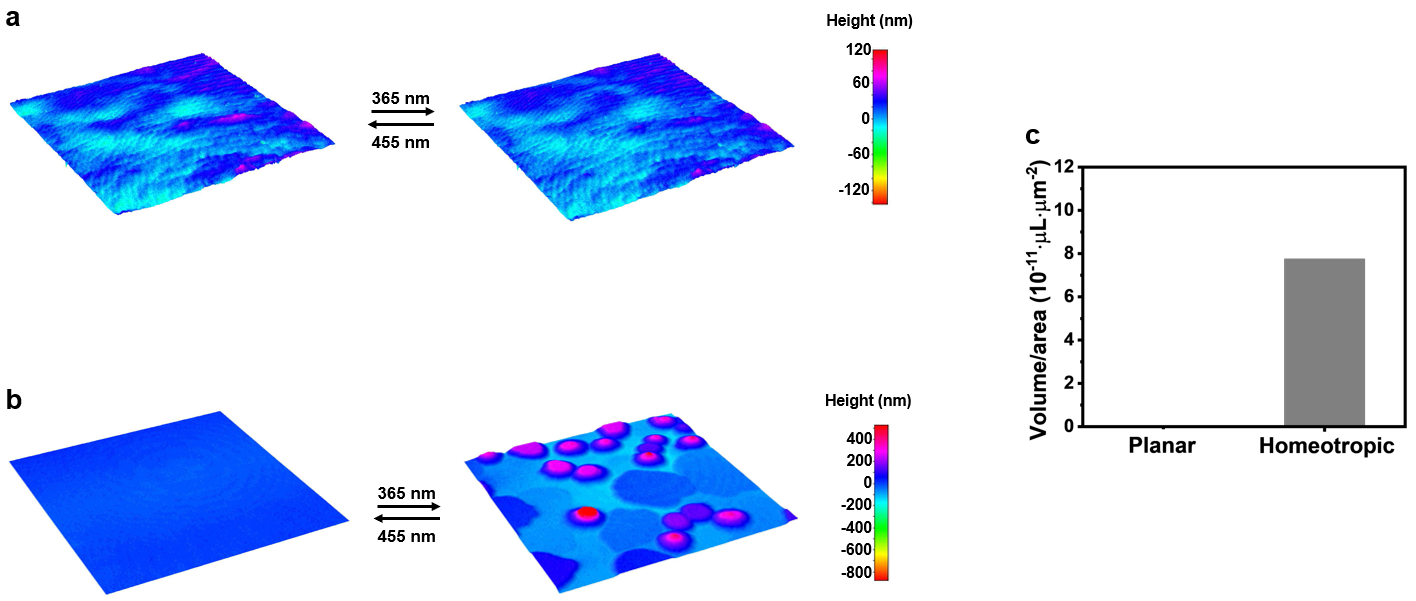


**Figure S4. Comparison of liquid secretion performance of the LCN coating with uniform planar or homeotropic alignment.** DHM characterization shows the liquid secretion performance of coating with a) uniform planar alignment and b) uniform homeotropic alignment, respectively. c) The secretion volume per unit area analyzed based on a) and b).


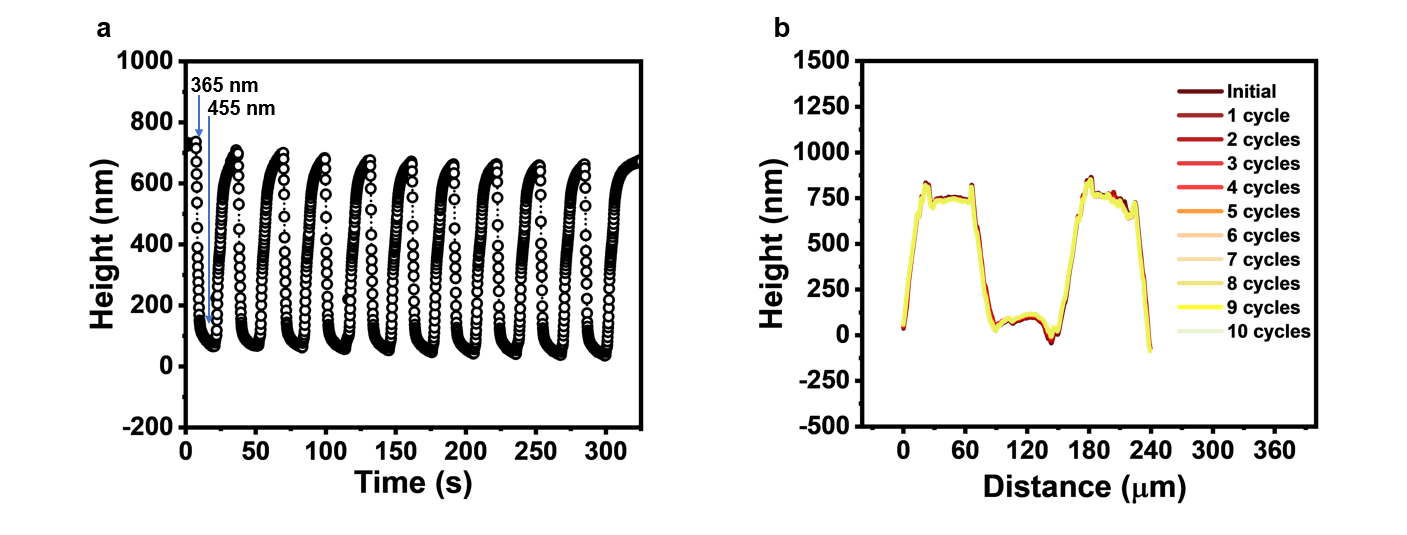


**Figure S5. Stability of the LCN coating as measured by multicycles of secretion and reabsorption process.** a) The surface height of the homeotropic region changes upon UV and blue light illumination, with 10 cycles of repeating. b) Coating surface profiles after each cycle of secretion and reabsorption.


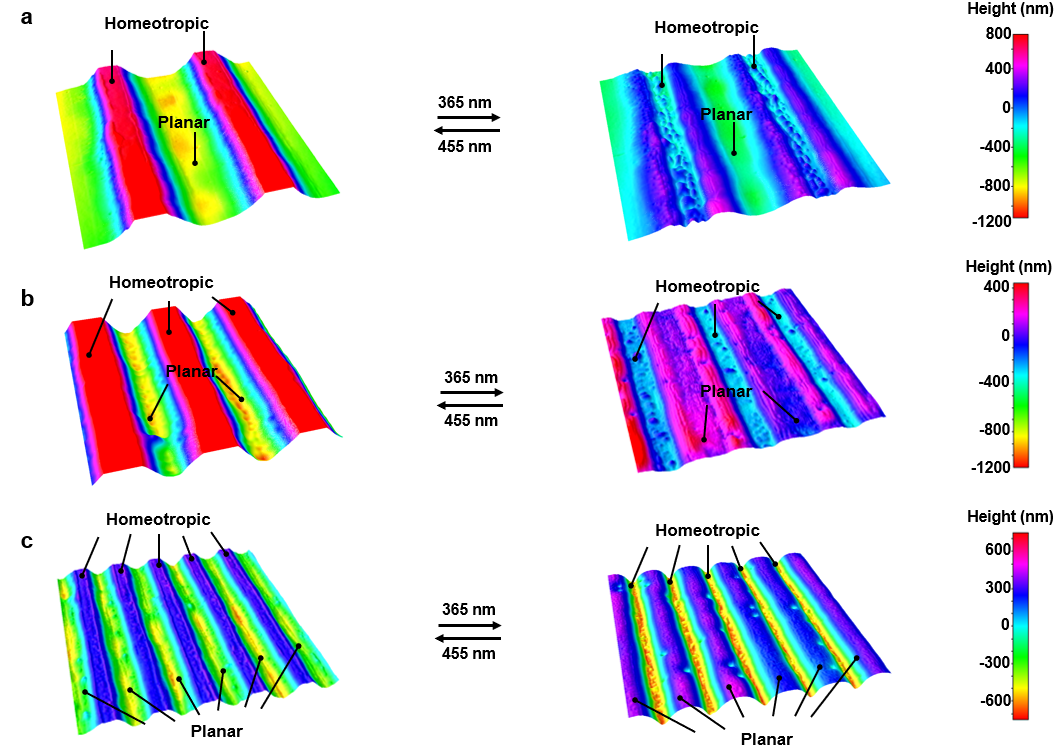


**Figure S6. Effect of electrode size on the secretion of the homeotropic-planar alternating LCN polymer coating.** The 3D surface profile of the coating electrically aligned by the electrode in widths of a) 30 μm, b) 20 μm, and c) 10 µm as stimulated by 365 nm and 455 nm light. The ratio of the electrode width to the gap width is kept at 1:3.

Table S1. Volume of localized secretion change as a function of electrode size

| Electrode size (µm) | Volume of Secreted liquid (10^-6^·µL) | Area of analyzed image (10^4^·µm^2^) | Area of secretion (10^4^·µm^2^) |
| --- | --- | --- | --- |
| 40 | 2.04 | 6.06 | 1.97 |
| 30 | 1.42 | 6.06 | 1.48 |
| 20 | 1.20 | 6.06 | 1.48 |
| 10 | 0.27 | 1.55 | 0.37 |

*The volume is analyzed based on images shown in Figure 3b and Figure S6.


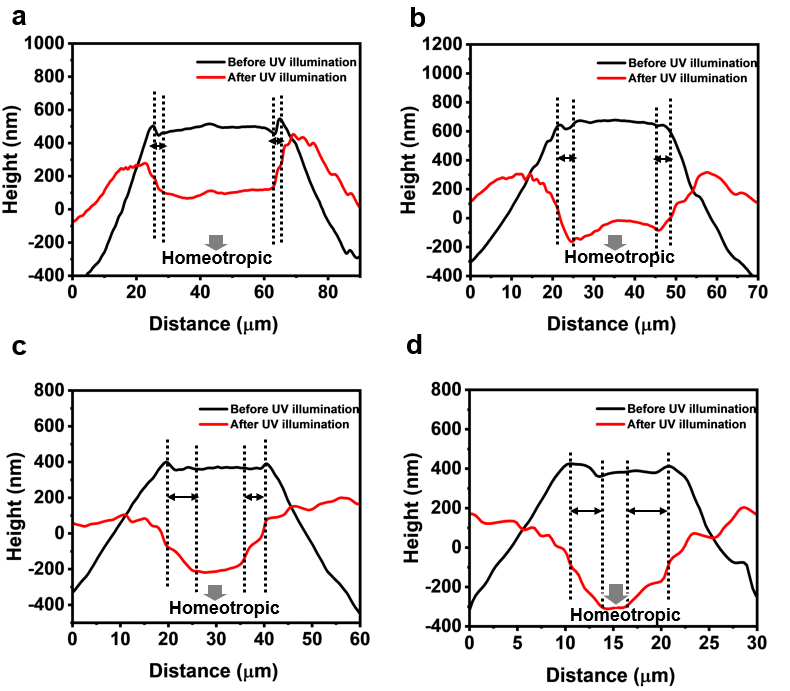


**Figure S7. Effect of electrode size on the transition area from homeotropic to planar of the LCN coating.** The curves show 2D profiles of the initial coatings that are aligned by the electrode with widths of a) 40 μm, b) 30 μm, c) 20, and d) 10 µm and the profiles after deformation upon UV illumination. By decreasing the electrode size from 40 µm to 10 µm, the ratio of the transition area to the electrode area increases from 4.1% to 15.9%, 51.5%, and 80%, respectively. The width of the transition area is calculated by subtracting the width of the homeotropic area after UV illumination from its width before UV illumination.


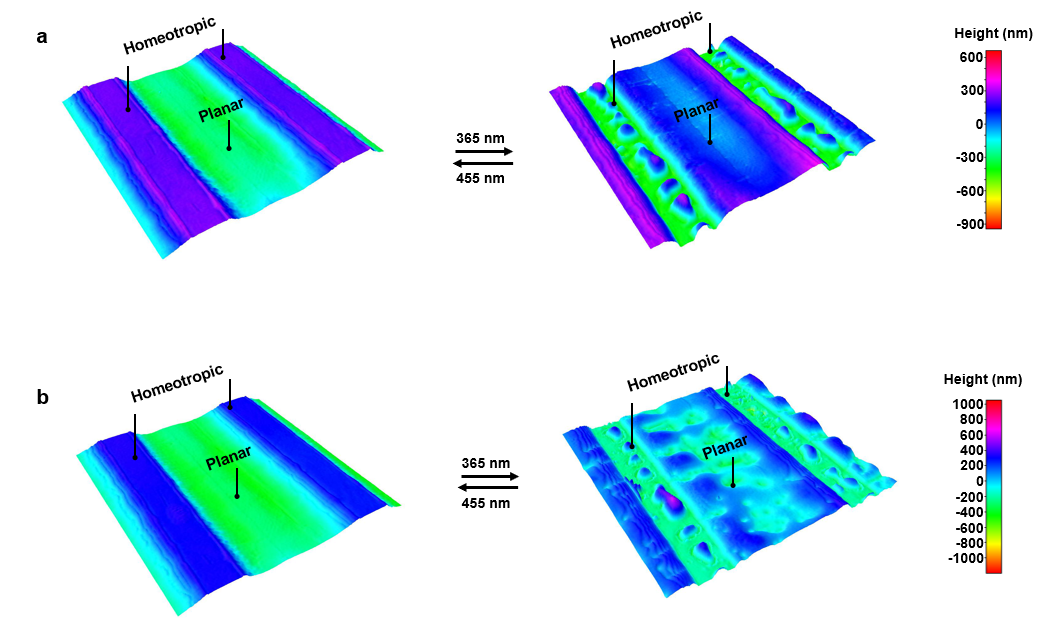


**Figure S8. Effect of temperature on the secretion of the homeotropic-planar alternating LCN polymer coating.** The 3D surface profile of the coating heated on the hotplate at the temperature of a) 35 °C and b) 41 °C as stimulated by 365 nm and 455 nm light.


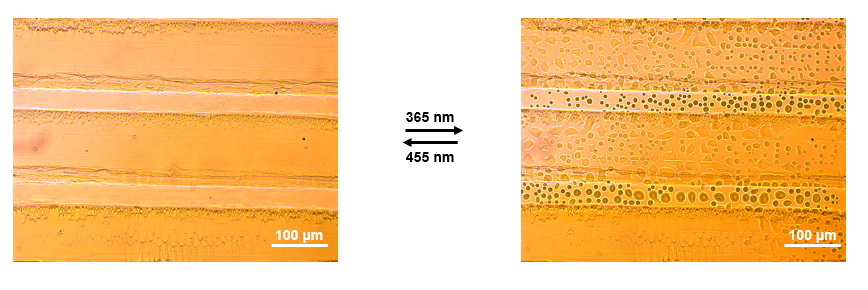


**Figure S9. Light-responsible secretion of the LCN coating as impacted by temperature.** Optical microscope images showing the coating stimulated by 365 nm and 455 nm light at 41 °C.


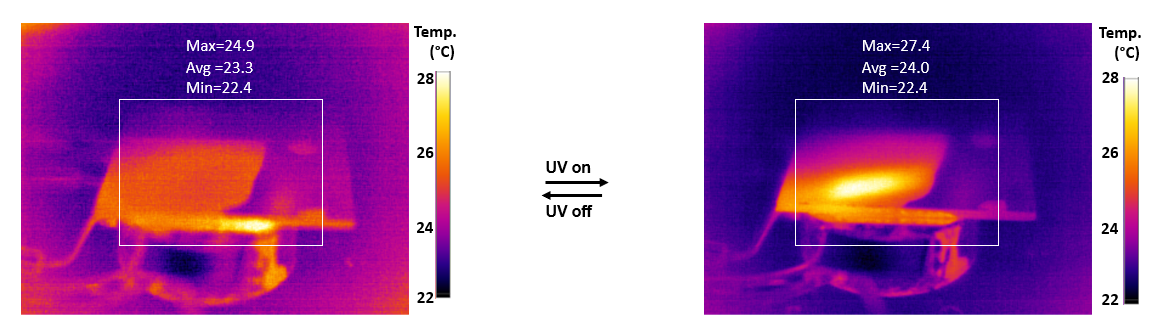


**Figure S10. Evaluation of UV thermal effect on the liquid secretion of the LCN coating.** Infrared camera images show the temperature of the coating change from 23.5 °C to 27.4 °C under UV illumination. The maximum temperature of the coating upon UV exposure doesn’t reach the phase transition temperature of 8CB, which would not be a trigger for secretion.


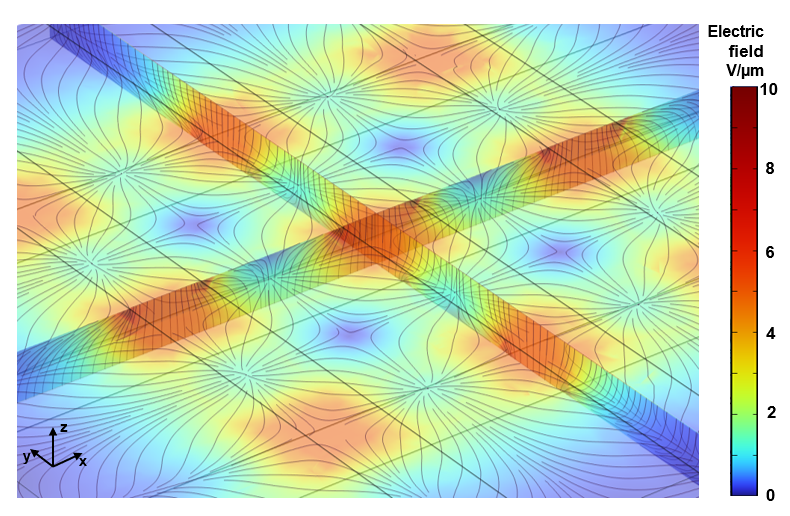


**Figure S11. Simulated 3D electric field distribution when the stripe electrodes at the top and the bottom are placed perpendicularly to each other.**
